# Epidemiological and Clinical Aspects of COVID-19; a Narrative Review 

**Published:** 2020-04-01

**Authors:** Goodarz Kolifarhood, Mohammad Aghaali, Hossein Mozafar Saadati, Niloufar Taherpour, Sajjad Rahimi, Neda Izadi, Seyed Saeed Hashemi Nazari

**Affiliations:** 1 Department of Epidemiology, School of Public Health & Safety, Shahid Beheshti University of Medical Sciences, Tehran, Iran.; 2 Modeling in Health Research Center, Shahrekord University of Medical Sciences, Shahrekord, Iran.; 3 Student Research Committee, Department of Epidemiology, School of Public Health and Safety, Shahid Beheshti University of Medical Sciences, Tehran, Iran.; 4 Prevention of Cardiovascular Disease Research Center, Department of Epidemiology, School of Public Health and Safety, Shahid Beheshti University of Medical Sciences, Tehran, Iran.

**Keywords:** COVID-19, severe acute respiratory syndrome coronavirus 2, epidemiology, public health, communicable diseases, emerging

## Abstract

There are significant misconceptions and many obstacles in the way of illuminating the epidemiological and clinical aspects of COVID-19 as a new emerging epidemic. In addition, usefulness of some evidence published in the context of the recent epidemic for decision making in clinic as well as public health is questionable. However, misinterpreting or ignoring strong evidence in clinical practice and public health probably results in less effective and somehow more harmful decisions for individuals as well as subgroups in general populations of countries in the initial stages of this epidemic. Accordingly, our narrative review appraised epidemiological and clinical aspects of the disease including genetic diversity of coronavirus genus, mode of transmission, incubation period, infectivity, pathogenicity, virulence, immunogenicity, diagnosis, surveillance, clinical case management and also successful measures for preventing its spread in some communities.

## Introduction

Over the past few decades, a large number of people have been affected with the 3 epidemics caused by coronavirus family (SARS-2003, MERS-2012, and COVID-2019) in the world. Nevertheless, there is substantial genetic dissimilarity between pathogens of the three previous epidemics, in particular MERS with COVID-19. In the previous epidemics, initial hotspots of diseases were Middle East, Saudi Arabia (MERS) and China and animal to human, and then human to human transmissions of pathogens were reported in other countries (1,2). 

For COVID-19, as suggested by epidemiological evidence in China (at the time of writing this paper), this outbreak began from a seafood and live animal shopping center in Wuhan, Hubei Province on December 12, 2019. However, similar to two previous epidemics, the current epidemic also switched to human to human transmission immediately, and swept through most regions in China even faster than the previous pandemics (3). 

Recent epidemics of viral respiratory diseases in the world have started from China (except for MERS that originated in Saudi Arabia), and there are several possible reasons for this. From an economic perspective, China has emerged as one of the leading countries in the production of various commodities, especially in the past decade, and given the enormous volume of trade, tourism and military transactions with other countries, there was no doubt that the virus would spread to other parts of the world (4).

China has already acknowledged the possibility of a new virus epidemic in the future and has consequently stressed the importance of formulating a policy to improve the healthcare system and preparedness after the two previous epidemics. This country rearranged its health plan in the wake of MERS epidemic in 2012, establish a new web-based service for quick alarming in case of an emerging disease with unknown origin through common surveillance system. In the wake of conditions ensuing SARS epidemic and severe criticisms levelled by international institutions regarding delayed provision and sharing of data by China government, this country has started extensive collaborations with international institutions from the early days of the recent epidemic, and established a publicly available database of line list of cases through coordinating with Johns Hopkins University (5). 

Moreover, China scaled up public health measures and quarantined many cities, bearing the grave economic consequences of this action to prevent the spread of the disease to other parts of the world. Although, China has been struggling with tough conditions in the previous month, reduction in the number of incidence cases and interruption of transmission indicate its successful measures to control the recent epidemic and highlight the importance of timely and appropriate decisions through activating human and material resources for addressing a serious global threat (6). 

Number of COVID-19 cases has risen substantially in the world compared to SARS and MERS, and it would probably take longer to halve the disease cases; meaning that control measures would have to be in place for a longer period of time. WHO has announced that Coronavirus epidemic is progressively increasing in three countries, including Italy, South Korea, and Iran. The shared string that links these three countries is the pandemic of MERS in 2013, which was transmitted through close human-to-human contacts (7). This study was carried out to review different epidemiological and clinical aspects of the new emerging disease along with specific measures by countries in the community level. 

## Methods

**Search methods and strategies for identification of studies**

Literature search was performed in “PubMed”, “Web of Science”, "Scopus", "ScienceDirect" and also in "JAMA", "BMJ", "Oxford" and "THE LANCET" journals using following terms: coronavirus, COVID-19 and 2019-nCoV, to find articles published from January 5 to February 28, 2020. Moreover, we used the findings of literature retrieved via searching authoritative texts and hand searches in WHO reports. We checked the reference lists of all studies identiﬁed by the above methods. Studies were excluded if used old data, had inappropriate topics and were not pertinent to the focused purpose of the study. 

**Data collection and analysis**

In order to identify studies meeting the inclusion criteria, seven review authors screened the titles and abstracts of all retrieved records. The studies were selected independently and the results were discussed to make the final selection. After reading the full text of all potentially eligible articles, a final decision was made for each study.

**Data extraction and management**

Extraction of data was performed by the same seven review authors who conducted the study selection independently, using a structured form that contained study characteristics including genetic diversity of coronavirus genus, mode of transmission, incubation period, infectivity, pathogenicity, virulence, immunogenicity, diagnosis, surveillance, clinical case management, special measures in community level and health care facility. Any disagreement was discussed after completion of data collection process and reviewers were consulted for each topic.

## Results

**Genetic differences between SARS, MERS, and COVID-19 epidemics**

The animal reservoir of the virus has not yet been identified, but genomic of COVID-19 is so similar to bat coronavirus (98%), reinforcing the presumption that the virus was transmitted by an animal in the shopping center in Wuhan. With regard to genomic similarity, the virus differs from its predecessors, namely SARS (79%) and MERS (50%). As indicated by genetic data, CVOID-19 pathogen is classified as a member of the beta-coronavirus genus, and can bind to the angiotensin-converting enzyme 2 receptor in humans (1,2).

**Transmission and Incubation period**

Human to human transmission via either respiratory droplets or close contacts was initially proposed as the main routes of transmission of the pathogen based on experience gained in the previous two epidemics caused by coronaviruses (MERS-CoV and SARS-CoV)(8). According to the world Health Organization (WHO) report, 2019-nCoV is a unique virus that causes respiratory disease, which spreads via oral and nasal droplets. Moreover, the pathogen of COVID-19 can float in the air in the form of aerosols and cause infection in healthy people (9). Evidence of a study in Singapore revealed higher loads of virus in confirmed cases of COVID-19 in early stages of the disease, which decreased dramatically over time (10). 

There is a limited number of evidence on oral-fecal transmissibility of the pathogen. However, COVID-19 RNA was found in fecal specimens of 2 to 10% of confirmed patients with gastrointestinal symptoms such as diarrhea (11,12), so fecal-oral transmission should be taken into account as a probable route through case investigation. 

Incubation period (the time from infection to the onset of symptoms) for the new pathogen varies from 2 to 14 days in human to human transmission (13). Furthermore, median incubation period was reported as 5-6 days (ranged from 0-14 days) in WHO report (14). Studies that were conducted on those who had traveled to Wuhan and Guangdong mean incubation period of 4.8 (±2.6) days was reported. In some other studies the mean incubation period was reported to be 6.4 days (15,16), while another study in China reported longer incubation times up to 24 days (13). 

**Infectivity**

An important question about COVID-19, which has raised much concern among health care providers, health policy makers and the general population, is the degree of transmissibility or contagiousness of the coronavirus (infectivity). In general, epidemiologists use mathematical formulas with clear and acceptable assumptions to calculate the infectivity index. For this purpose, "basic reproduction number" termed R_0_ is used, and it indicates the expected number of cases directly infected by one contagious case in a population that everyone is supposed to be susceptible. For viral pathogens in MERS and SARS epidemics, the index was approximated to be 2, indicating that each infected person could infect two people on average in an effective contact. However, for COVID-19, the calculated value in a study was slightly higher and the index value based on data calculated in Wuhan, China was 2.2 (95% CI, 1.4 to 3.9) (17) and it shows that the infectivity of COVID-19 is higher than previous epidemics originated by coronavirus (18). In other studies, R_0_ has been reported with different values, the lowest of which corresponds to the WHO report of 1.95 (1.4-2.5) (19) and the highest value is 6.47 (95% CI 5.71–7.23) (20). A review study estimated an average R_0_ for COVID-19 of 3.28 with a median of 2.79 and an IQR of 1.16 (21). As an explanation for variety of the calculated indices is that different calculation methods were used and calculations were done at different times of epidemics. 

As previously noted, certain assumptions have been made in calculation of this index. Initial reports on a family in one of the provinces of China show that all six-members of a family, aged 10-66 years were infected within a short period after one member returned from Wuhan (8). As a conclusion, this index is changing over time, and its reduction may reflect effectiveness of preventive measures, so that reaching a value less than one (less than one new case per effective contact with an infected person and transmission) implies that the epidemic is controlled in the community (6).

**Pathogenicity**

An important concern in the recent pandemic is the capability of the pathogen to establish and induce infection with different clinical manifestations in human. According to WHO report, about 82 percent of COVID-19 patients have mild symptoms and were recovered immediately. As of 20 February, there were 18264 (24%) recovered cases in China and recovery and mortality rates of the disease among severe cases in Guangdong were 26.4 % and 13.4%, respectively. Median time for onset of symptoms to recovery in mild and severe cases was 2 and 3-6 weeks, respectively. Furthermore, time interval between onset and developing severe symptoms such as hypoxia was one week (22).

In case studies that were conducted outside of mainland China, time of onset of symptom(s) to recovery was 22.2 days (95% confidence interval 18-83). Moreover, average time of onset of symptom(s) to death varies from 20.2 (95% confidence interval 15.1-29.9) to 22.3 days (95% confidence interval 18-82) (23,24). Results of a case-series study on six infants (45-days to one-year) infected with COVID-19 in China indicated mild symptoms of the disease in this age group with no need for further intensive care (25). According to WHO report, COVID-19 disease among children seems to be rare with mild symptoms, about 2.4% of total cases were reported in children and adolescents (aged under 19 years), while older cases aged over 60 years and those with a background of chronic diseases were at higher risk of developing severe disease and death (22). 

Even though age is an important deterministic factor for severity of symptoms, other risk factors such as having a history of underlying diseases and/or co-infection with other infections like Influenza virus and Klebsiella may accelerate the progress of symptoms and lead to poor prognosis of the disease (26). However, findings from a study in Singapore shows that infected patients with no history of underlying diseases may also develop severe disease and need for intensive care (4). 

**Virulence**

The virulence of a disease is usually measured on the basis of indicators such as mortality rate and disability. Compared with the previous two epidemics (SARS and MERS), the case fatality rate was lower and approximately 2% in COVID-19, and only less than 15% of patients would seek hospital services. However, the case fatality rate of SARS and MERS was 10% and 34%, respectively (18). Results of a study in China revealed the overall case fatality rate of 2.3% for COVID-19 (27) and some studies reported case fatality rate of 0.9% in Beijing (28). In another study, Jung and colleagues reported a confirmed case fatality risk of 5.3% to 8.4% for COVID-19(23). However, due to the rapid spread of COVID-19, there is a higher number of death cases in the recent pandemic (N=3043, up to 02 March 2020) compared to SARS and MERS (N=1871) (29).

There is a poor prognosis for the disease in middle and older aged patients (28). In a study on 44672 confirmed cases in China, case fatality rate was highest in the group of over 80 years (14.77%), followed by the age group between 70 to 80 years (7.96%) and no mortality was reported in age group below 10 years (30). Even though death outcome is uncommon in young people, a few deaths are reported in this age group in China and Iran. 

Availability of and access to healthcare facilities has likely contributed to increase in death outcome. As a probable explanation for the difference between fatality rate in Wuhan (3%) and other provinces (0.7%) in China, death rates are likely affected by shortage in health resources due to increasing number of patient who had sought diagnosis and treatment services in the early phase of the epidemic in Wuhan (31).

**Immunogenicity**

Exploring and understanding the immunogenicity of COVID-19 is essential for developing the most effective treatment regimens and vaccine. However, evidence on immunogenicity of COVID-19 is limited. Study on B-cell and T-cells epitopes revealed that SARS-CoV and the virus causing COVID-19 had identical proteins (32). A few clinical trials have evaluated the efficacy of new vaccines in MERS-CoV and SARS-CoV. Results of these studies in Phase-1 showed some degree of efficacy and one of these studies has been certified to begin Phase-2 (33,34). 

Absence of clinical symptoms, respiratory lesions in CT scan and two negative RT-PCR tests in two consecutive days are introduced as criteria of discharge from hospital or quarantine center in China (35). However, recent studies reported several cases of COVID-19 with clinical manifestations of the disease along with a positive test after discharging from hospital (36,37). False positive and false negative results have been reported in RT-PCR test (10,38); hence, hospitals in China have considered additional antibody test (negative IgM and positive IgG results) as a recovery criteria and discharge requirement (39). In conclusion, recurrence of COVID-19 in recovered cases highlights the necessity for development of a more effective vaccine. 

**Diagnosis**

Pathogen of COVID-19 has been detected in upper and lower respiratory tracts in initial assessments. Moreover, viral RNA has been detected in fecal and blood samples in later studies. According to WHO guideline, laboratory diagnosis of COVID-19 is based on a positive RT-PCR test. Target gene for diagnosis may be different by country. Accordingly, target genes for screening and confirmatory assays by RT-PCR are ORF1ab and N in Chinese laboratory protocol, while RdRP, E and N are checked in Germany. Furthermore, three targets in N gene are considered in the US protocol (40). 

RT-PCR is an expensive test and no access to diagnostic facility during COVID-19 pandemic advocates conducting new researches on other diagnostic approaches such as Chest CT. However, results of recent studies in China demonstrate low specificity for this diagnostic approach (41). As a critical point in diagnostic studies, accuracy of a new test is compared to the gold standard. This comparison resulted in lower values of diagnostic accuracy for the new test. On the other hand, the sensitivity and specificity of a test depend on the severity of cases, which may vary between different populations according to their type of surveillance system (42). In the mentioned study that compared CT scan with RT-PCR as a gold standard, sensitivity of CT scan was appropriate (41). However, the study population consisted of suspected cases and generalizability of the findings is questionable (43). Furthermore, the large number of hospitalized cases due to false positive results by CT scan may increase the risk of transmission to healthy people. On the other hand, RT-PCR test may be subject to some limitations, especially in the earlier phase of an epidemic, as the specialists should be trained for running related procedures and interpretation of results. Moreover, false negative results due to either low quality of specimen in use or inadequate number of organisms in the samples are introduced as main challenges (44). Results of a recent study on rapid IgM-IgG combined test revealed some limitation for RT-PCR test as a standard diagnostic method for COVID-19. The following limitations were indicated for RT-PCR test: long turnaround times, complex operation, and need for quality controlled laboratories, expensive equipment and trained specialists (38). 

**Surveillance ** The outbreak surveillance is the anticipation, early warning, prompt detection and response to unusual increase in the number of cases. Establishing a surveillance system for a new epidemic is believed to be a core intervention in controlling the disease (45). Surveillance system data provides reliable information for epidemiologists to identify weak chains of transmission and facilitates evidence-based decisions by policymakers both inside and outside the healthcare service. Moreover, updating and sharing interpretations of data with media, especially in earlier phase of an epidemic, will aid community engagement and participation in control activities and prevention of spreading rumors. Although, it may be too soon to compare the effectiveness of surveillance systems for COVID-19 epidemic in different countries, it seems that the Chinese surveillance system is highly effective as it ensures timely detection, recording, tracking, updating and sharing information on media for an outbreak with unknown origin and high burden of cases (4).

In a large number of countries, the initial focus of the surveillance system for CIVID-19 is examination of all suspected cases with symptoms of the disease (mostly fever) and all people with a travel history to China or visiting Chinese travelers or citizens it the previous two weeks. However, this type of screening program mainly relies on fever cases and those with direct flights from China, so it misses pre-symptomatic cases as well as infected travelers who are arriving from regions with high burden of disease via indirect flights, which could be a source of infection in COVID-19-free countries (46).

In a communicable disease outbreak, essential data are usually collected in parallel from different available information sources in the country including data of weekly outpatient visits to health care centers and hospital referrals with a chief complaint of fever, data of weekly inpatient fever cases and deaths with unknown origin (45). Furthermore, increase in the number of cases and deaths due to pneumonia may raise an alarm in COVID-19 free areas. 

A prerequisite for establishing a surveillance system is to provide basic laboratory facilities, particularly at "point of care" (10). This system should be constantly monitored and evaluated using sensitive indicators to ensure the quality of case detection, diagnosis and management. 

Detection of primary confirmed cases with poor prognosis in early phase of the epidemic without any link to confirmed cases from other regions emphasis on the insensitivity of a national and local surveillance systems and low performance of control activities against the disease in community level. In this case, it should be immediately addressed and capability and capacity of the surveillance system should be checked. 

**Examples of surveillance systems in different countries (** 47 **)**

National authorities are actively looking for cases in all provinces of China and efforts for finding additional cases inside and outside of Wuhan City have been expanded. Moreover, active and reactive case detection along with tracing close contacts have been started in medical institutions. 

The Department of Disease Control in Thailand scaled up the Emergency Operations Center to Level 3 to closely monitor the ongoing situation in both national and international levels. This country has started a screening program to check for fever in all travelers who arrived from Wuhan through direct flights in airports. 

Japan’s Ministry of Health requested local health governments to be aware of the respiratory illnesses in Wuhan using the existing surveillance system for serious infectious illnesses with unknown etiology. It has strengthened surveillance for undiagnosed severe acute respiratory illnesses. Quarantine and screening measures have been intensified for travelers from Wuhan at the points of entry. Furthermore, National Institute of Infectious Disease (NIID) established an in-house PCR assay for COVID-19. 

Contact tracing and other epidemiological investigation are ongoing in the Republic of Korea to prevent the spread of the disease. The government has scaled up the national alert level from Blue (Level 1) to Yellow (Level 2 of the 4-level national crisis management system). Surveillance of pneumonia cases has been strengthened in health facilities nationwide and quarantine and screening measures have been enhanced for travelers from Wuhan at the points of entry. 

The US centers for disease control and prevention (CDC) activated its Emergency Response System to provide ongoing support against COVID-19. Screening of passengers on direct and indirect flights from Wuhan China to the 3 main ports of entry in the United States has begun and will expand to Atlanta and Chicago in the coming days. CDC deployed a team to support ongoing investigation in the state of Washington and tracing close contacts following the first reported case of COVID-19.

**Clinical Case Management**

Diagnosis of COVID-19 based on clinical manifestations is complicated and initial symptoms of the disease are usually nonspecific. A large number of patients present to clinics and health centers with mild common cold symptoms such as dry cough, sore throat, low-grade fever or body aches. Patients usually go to the emergency departments if the symptoms of the clinical manifestations worsen after a few days. Because of the wide spectrum of clinical symptoms, research on biomarkers and clinical criteria predicting prognosis is of high priority to enable differentiating cases that require further interventions in the early phase of the disease (10).

No approved drug regimen has been introduced to treat infected cases so far, antiviral treatments are used to alleviate the disease symptoms. Studies on Remedesevir, as an antiviral agent, revealed its in vitro activity against the COVID-19 virus and its safety was proven in Ebola trials. Another proposed treatment is Chloroquine, an old drug for treatment of malaria, with apparent effectiveness and acceptable safety against COVID-19 associated pneumonia (48,49). Evaluating the efficacy of anti-influenza drugs such as Umifenovir and Oseltamivir against COVID-19 virus is interesting but lacks any biological plausibility. Using monoclonal antibodies has been suggested as an attractive choice among inactive prophylactic methods; however, its effectiveness has not been proven in other viral respiratory diseases and influenza, yet (50,51).

Steroids and methylprednisolone seem to be widely used in the recent pandemic. However, in case of MERS, it has been shown that the drug prolongs the presence of the virus and WHO does not recommend its use for COVID-19, except for patients with acute respiratory distress syndrome (ARDS) (52,53). 

The effectiveness of other medicines and regimens such as Chloroquine, Vitamin C, and Chinese medicine, as well as Lopinavir/Ritonavir combination therapy and Remedesevir are being evaluated in China. Even though randomized clinical trials are important for improving prognosis and interrupting transmission of disease, researchers and healthcare providers should concentrate on alleviation of the disease among subgroups of patients and in different phases of the disease (54).

In addition, since the emerging virus has become a serious global concern, there is a need for rapid development of a vaccine. There are a few vaccine candidates developed in response to outbreak. However, an effective anti-viral medication or a vaccine that has been evaluated for safety and efficacy against COVID-19 is not available yet, and most vaccines are still in the preclinical testing stage (55,56,57). 

**Special intervention in community level**

So after the rise of an emerging disease, goverments have a special responsibility to balance between civil liberties and special measures for protecting susceptible populations (46). However, three components of "scientific", "voluntary" and civil liberty should be considered as guiding principles for decision-making and operating each special protective measure at the community level. Through their experiences in previous communicable disease epidemics, US public health authorities found that enhanced screening programs, monitoring healthy people and quarantine at the community level were not effective measures against progressive spreading of disease. Therefore, specific regulations and waivers were declared to prevent traveling to mainland china and flights to and from China were temporarily suspended. Passengers and US citizens with a history of traveling to China during the previous month were encouraged to stay home and self-quarantine for up to 14 days. However, these interventions and recommendations were deemed insufficint, so public health experts warned about expanding transmission throughout the country in the coming weeks as a consequence of population movements and large scale spread of the disease all over the world (46,58). 

Even though children are important sources of influenza virus transmission in the community, initial data analysis on COVID-19 indicated that children were mainly infected from adults rather than the other way around. However, clinical attack rates are low in children and teenagers (0-19) (59), so this age-group may contribute to continuous transmission in the communty. Therefore, countries with high prevalnece of the disease, such as China, Iran, Italy, South korea and Japan, closed or postponed the start of school and extended holidays. 

Other special measures considered for control of the pandemic at community level include: cancellin mass gatherings, religious services, tourism, cultural and sport events, concerts and other events. In the mentioned countries, healthcare authorities issued travel ban to and from affected areas and allowed non-essential personnel and employees to work from home. 

**Special interventions for healthcare providers**

Healthcare authorities are responsible for predicting and supplying the essential protective equipment for general population as well as healthcare providers. By ensuring their availability through effective supply chain management, they gain public trust. They also have to plan for deploying healthcare perssonel from less affected areas to epidemic regions (10). With this method, a large number of medical staff and nurses were voluntarily deployed to Wuhan, China (60).

According to primary reports from China and Singapre, working with protective equipment for a long time is cumbersome for healthcare providers and they are under tremendous stress due to probability of being infection and transmitting the disease to their families through close contact (57). The high rates of hospital infection in the recent pandemic emphasizes the importance of regular examination for symptoms among healthcare providers who are in close contact with confirmed patients in order to isolate them in case of positive laboratory test. 

## Conclusion

COVID-19 pandemic is a major international test for the medical community, revealing weaknesses in management of emerging viral diseases and reminding us that communicable diseases must never be underestimated or dealt with using insufficient resources. The present situation also enables governments to evaluate their capabilities and capacities to organize human and material resources, share and analyze data in a timely manner and cooperate with media, journalists and local communities to implement control activities. 
